# Supplementary material for: Barriers and Facilitators to the Implementation of Effective Alcohol Control Policies: A Scoping Review
Source: Int J Environ Res Public Health. 2022 May 31;19(11):6742. doi: 10.3390/ijerph19116742 (PMC9180061; doi:10.3390/ijerph19116742)
Supplement: Supplementary file 1 [file ijerph-19-06742-s001.zip › supplementary_Table S1.pdf]

Supplementary

Table S1: Search terms across different databases

Search terms	Number of studies found
Scopus	
((TITLE-ABS (alcohol* W/10 polic*)) AND (TITLE-ABS-KEY(implement* OR enforc* OR monitor* OR compliance))) OR ((TITLE-ABS (alcohol* W/10 regulat*)) AND (TITLE-ABS-KEY(implement* OR enforc* OR monitor* OR compliance))) OR ((TITLE-ABS (alcohol* W/10 strateg*)) AND (TITLE-ABS-KEY(implement* OR enforc* OR monitor* OR compliance))) OR ((TITLE-ABS (alcohol* W/10 law*)) AND (TITLE-ABS-KEY(implement* OR enforc* OR monitor* OR compliance))) OR ((TITLE-ABS (alcohol* W/10 legislation)) AND (TITLE-ABS-KEY(implement* OR enforc* OR monitor* OR compliance))) AND NOT (TITLE-ABS ("non-alcohol*" OR "brief screening" OR screening OR "alcohol treatment")) AND NOT SUBJAREA (vete) AND (LIMIT-TO (PUBYEAR , 2021) OR LIMIT-TO (PUBYEAR , 2020) OR LIMIT-TO (PUBYEAR , 2019) OR LIMIT-TO (PUBYEAR , 2018) OR LIMIT-TO (PUBYEAR , 2017) OR LIMIT-TO (PUBYEAR , 2016) OR LIMIT-TO (PUBYEAR , 2015) OR LIMIT-TO (PUBYEAR , 2014) OR LIMIT-TO (PUBYEAR , 2013) OR LIMIT-TO (PUBYEAR , 2012) OR LIMIT-TO (PUBYEAR , 2011) OR LIMIT-TO (PUBYEAR , 2010) OR LIMIT-TO (PUBYEAR , 2009) OR LIMIT-TO (PUBYEAR , 2008) OR LIMIT-TO (PUBYEAR , 2007) OR LIMIT-TO (PUBYEAR , 2006) OR LIMIT-TO (PUBYEAR , 2005) OR LIMIT-TO (PUBYEAR , 2004) OR LIMIT-TO (PUBYEAR , 2003) OR LIMIT-TO (PUBYEAR , 2002) OR LIMIT-TO (PUBYEAR , 2001) OR LIMIT-TO (PUBYEAR , 2000)) AND (LIMIT-TO (DOCTYPE , "ar") OR LIMIT-TO (DOCTYPE , "re") OR LIMIT-TO (DOCTYPE ,	1904

Search terms	Number of studies found
"cp") OR LIMIT-TO (DOCTYPE , "sh")) AND (LIMIT-TO (LANGUAGE , "English"))	
(TITLE-ABS (taxes OR tax OR taxation OR pric* OR affordability OR "minimum pricing" OR "minimum unit pricing" OR "floor price")) AND (TITLE-ABS (alcohol*)) AND (TITLE-ABS-KEY(implement* OR enforc* OR monitor* OR compliance)) AND NOT "non-alcohol*" AND NOT SUBJAREA (vete) AND (LIMIT-TO (PUBYEAR , 2021) OR LIMIT-TO (PUBYEAR , 2020) OR LIMIT-TO (PUBYEAR , 2019) OR LIMIT-TO (PUBYEAR , 2018) OR LIMIT-TO (PUBYEAR , 2017) OR LIMIT-TO (PUBYEAR , 2016) OR LIMIT-TO (PUBYEAR , 2015) OR LIMIT-TO (PUBYEAR , 2014) OR LIMIT-TO (PUBYEAR , 2013) OR LIMIT-TO (PUBYEAR , 2012) OR LIMIT-TO (PUBYEAR , 2011) OR LIMIT-TO (PUBYEAR , 2010) OR LIMIT-TO (PUBYEAR , 2009) OR LIMIT-TO (PUBYEAR , 2008) OR LIMIT-TO (PUBYEAR , 2007) OR LIMIT-TO (PUBYEAR , 2006) OR LIMIT-TO (PUBYEAR , 2005) OR LIMIT-TO (PUBYEAR , 2004) OR LIMIT-TO (PUBYEAR , 2003) OR LIMIT-TO (PUBYEAR , 2002) OR LIMIT-TO (PUBYEAR , 2001) OR LIMIT-TO (PUBYEAR , 2000)) AND (LIMIT-TO (DOCTYPE , "ar") OR LIMIT-TO (DOCTYPE , "re") OR LIMIT-TO (DOCTYPE , "cp") OR LIMIT-TO (DOCTYPE , "bz") OR LIMIT-TO (DOCTYPE , "sh")) AND (LIMIT-TO (LANGUAGE , "English"))	407
(TITLE-ABS (sale* OR “trading hour*” OR “intoxicat*” OR "on-premis*" OR "off-premis*" OR "minimum age" OR "minimum legal age" OR availabilit* OR retail* OR outlet* OR licen* OR "online deliver*" OR "online sale*")) AND (TITLE-ABS (alcohol*)) AND (TITLE-ABS-KEY(implement* OR enforc* OR monitor* OR compliance)) AND NOT "non-alcohol*" AND NOT SUBJAREA (vete) AND (LIMIT-TO (PUBYEAR , 2021) OR	2,189

Search terms	Number of studies found
LIMIT-TO (PUBYEAR , 2020) OR LIMIT-TO (PUBYEAR , 2019) OR LIMIT-TO (PUBYEAR , 2018) OR LIMIT-TO (PUBYEAR , 2017) OR LIMIT-TO (PUBYEAR , 2016) OR LIMIT-TO (PUBYEAR , 2015) OR LIMIT-TO (PUBYEAR , 2014) OR LIMIT-TO (PUBYEAR , 2013) OR LIMIT-TO (PUBYEAR , 2012) OR LIMIT-TO (PUBYEAR , 2011) OR LIMIT-TO (PUBYEAR , 2010) OR LIMIT-TO (PUBYEAR , 2009) OR LIMIT-TO (PUBYEAR , 2008) OR LIMIT-TO (PUBYEAR , 2007) OR LIMIT-TO (PUBYEAR , 2006) OR LIMIT-TO (PUBYEAR , 2005) OR LIMIT-TO (PUBYEAR , 2004) OR LIMIT-TO (PUBYEAR , 2003) OR LIMIT-TO (PUBYEAR , 2002) OR LIMIT-TO (PUBYEAR , 2001) OR LIMIT-TO (PUBYEAR , 2000)) AND (LIMIT-TO (DOCTYPE , "ar") OR LIMIT-TO (DOCTYPE , "re") OR LIMIT-TO (DOCTYPE , "cp") OR LIMIT-TO (DOCTYPE , "sh") OR LIMIT-TO (DOCTYPE , "bz")) AND (LIMIT-TO (LANGUAGE , "English"))	
(TITLE-ABS (marketing OR promot* OR adverti* OR sponsor*)) AND (TITLE-ABS (alcohol*)) AND (TITLE-ABS-KEY(implement* OR enforc* OR monitor* OR compliance)) AND NOT "non-alcohol*" AND NOT SUBJAREA (vete) AND (LIMIT-TO (PUBYEAR , 2021) OR LIMIT-TO (PUBYEAR , 2020) OR LIMIT-TO (PUBYEAR , 2019) OR LIMIT-TO (PUBYEAR , 2018) OR LIMIT-TO (PUBYEAR , 2017) OR LIMIT-TO (PUBYEAR , 2016) OR LIMIT-TO (PUBYEAR , 2015) OR LIMIT-TO (PUBYEAR , 2014) OR LIMIT-TO (PUBYEAR , 2013) OR LIMIT-TO (PUBYEAR , 2012) OR LIMIT-TO (PUBYEAR , 2011) OR LIMIT-TO (PUBYEAR , 2010) OR LIMIT-TO (PUBYEAR , 2009) OR LIMIT-TO (PUBYEAR , 2008) OR LIMIT-TO (PUBYEAR , 2007) OR LIMIT-TO (PUBYEAR , 2006) OR LIMIT-TO (PUBYEAR , 2005) OR LIMIT-TO (PUBYEAR , 2004) OR LIMIT-TO (PUBYEAR , 2003) OR LIMIT-TO (	1,982

Search terms	Number of studies found
PUBYEAR , 2002) OR LIMIT-TO (PUBYEAR , 2001) OR LIMIT-TO (PUBYEAR , 2000)) AND (LIMIT-TO (DOCTYPE , "ar") OR LIMIT-TO (DOCTYPE , "re") OR LIMIT-TO (DOCTYPE , "cp") OR LIMIT-TO (DOCTYPE , "sh") OR LIMIT-TO (DOCTYPE , "bz")) AND (LIMIT-TO (LANGUAGE , "English"))	
(TITLE-ABS (driv* OR "blood alcohol concentration")) AND (TITLE-ABS (alcohol*)) AND (TITLE-ABS-KEY (implement* OR enforc* OR monitor* OR compliance)) AND NOT "non-alcohol*" AND NOT SUBJAREA (vete) AND (LIMIT-TO (PUBYEAR , 2021) OR LIMIT-TO (PUBYEAR , 2020) OR LIMIT-TO (PUBYEAR , 2019) OR LIMIT-TO (PUBYEAR , 2018) OR LIMIT-TO (PUBYEAR , 2017) OR LIMIT-TO (PUBYEAR , 2016) OR LIMIT-TO (PUBYEAR , 2015) OR LIMIT-TO (PUBYEAR , 2014) OR LIMIT-TO (PUBYEAR , 2013) OR LIMIT-TO (PUBYEAR , 2012) OR LIMIT-TO (PUBYEAR , 2011) OR LIMIT-TO (PUBYEAR , 2010) OR LIMIT-TO (PUBYEAR , 2009) OR LIMIT-TO (PUBYEAR , 2008) OR LIMIT-TO (PUBYEAR , 2007) OR LIMIT-TO (PUBYEAR , 2006) OR LIMIT-TO (PUBYEAR , 2005) OR LIMIT-TO (PUBYEAR , 2004) OR LIMIT-TO (PUBYEAR , 2003) OR LIMIT-TO (PUBYEAR , 2002) OR LIMIT-TO (PUBYEAR , 2001) OR LIMIT-TO (PUBYEAR , 2000)) AND (LIMIT-TO (DOCTYPE , "ar") OR LIMIT-TO (DOCTYPE , "re") OR LIMIT-TO (DOCTYPE , "cp") OR LIMIT-TO (DOCTYPE , "sh")) AND (LIMIT-TO (LANGUAGE , "English"))	1,778
(((TITLE-ABS (alcohol* W/10 polic*)) AND (TITLE-ABS-KEY (implement* OR enforc* OR monitor* OR compliance))) OR ((TITLE-ABS (alcohol* W/10 regulat*)) AND (TITLE-ABS-KEY (implement* OR enforc* OR monitor* OR compliance))) OR ((TITLE-ABS (alcohol* W/10 strateg*)) AND (TITLE-ABS-KEY (implement* OR enforc* OR	6,102

Search terms	Number of studies found
monitor* OR compliance))) OR ((TITLE-ABS (alcohol* W/10 law*)) AND (TITLE-ABS-KEY (implement* OR enforc* OR monitor* OR compliance))) OR ((TITLE-ABS (alcohol* W/10 legislation)) AND (TITLE-ABS-KEY (implement* OR enforc* OR monitor* OR compliance))) AND NOT (TITLE-ABS ("non-alcohol*" OR "brief screening" OR screening OR "alcohol treatment")) AND NOT SUBJAREA (vete)) OR ((TITLE-ABS (taxes OR tax OR taxation OR pric* OR affordability OR "minimum pricing" OR "minimum unit pricing" OR "floor price")) AND (TITLE-ABS (alcohol*)) AND (TITLE-ABS-KEY (implement* OR enforc* OR monitor* OR compliance)) AND NOT "non-alcohol*" AND NOT SUBJAREA (vete)) OR ((TITLE-ABS (sale* OR "trading hour*" OR "intoxicat*" OR "on-premis*" OR "off-premis*" OR "minimum age" OR "minimum legal age" OR availabilit* OR retail* OR outlet* OR licen* OR "online deliver*" OR "online sale*")) AND (TITLE-ABS (alcohol*)) AND (TITLE-ABS-KEY (implement* OR enforc* OR monitor* OR compliance)) AND NOT "non-alcohol*" AND NOT SUBJAREA (vete)) OR ((TITLE-ABS (marketing OR promot* OR adverti* OR sponsor*)) AND (TITLE-ABS (alcohol*)) AND (TITLE-ABS-KEY (implement* OR enforc* OR monitor* OR compliance)) AND NOT "non-alcohol*" AND NOT SUBJAREA (vete)) OR ((TITLE-ABS (driv* OR "blood alcohol concentration")) AND (TITLE-ABS (alcohol*)) AND (TITLE-ABS-KEY (implement* OR enforc* OR monitor* OR compliance)) AND NOT "non-alcohol*" AND NOT SUBJAREA (vete)) AND (LIMIT-TO (LANGUAGE , "English")) AND (LIMIT-TO (DOCTYPE , "ar") OR LIMIT-TO (DOCTYPE , "re") OR LIMIT-TO (DOCTYPE , "cp") OR LIMIT-TO (DOCTYPE , "sh") OR LIMIT-TO (DOCTYPE , "bz")) AND (LIMIT-TO (PUBYEAR , 2021) OR LIMIT-TO (PUBYEAR , 2020) OR LIMIT-TO (PUBYEAR , 2019) OR LIMIT-TO (PUBYEAR , 2018) OR LIMIT-TO (	

Search terms	Number of studies found
PUBYEAR , 2017) OR LIMIT-TO (PUBYEAR , 2016) OR LIMIT-TO (PUBYEAR , 2015) OR LIMIT-TO (PUBYEAR , 2014) OR LIMIT-TO (PUBYEAR , 2013) OR LIMIT-TO (PUBYEAR , 2012) OR LIMIT-TO (PUBYEAR , 2011) OR LIMIT-TO (PUBYEAR , 2010) OR LIMIT-TO (PUBYEAR , 2009) OR LIMIT-TO (PUBYEAR , 2008) OR LIMIT-TO (PUBYEAR , 2007) OR LIMIT-TO (PUBYEAR , 2006) OR LIMIT-TO (PUBYEAR , 2005) OR LIMIT-TO (PUBYEAR , 2004) OR LIMIT-TO (PUBYEAR , 2003) OR LIMIT-TO (PUBYEAR , 2002) OR LIMIT-TO (PUBYEAR , 2001) OR LIMIT-TO (PUBYEAR , 2000))	
Web of Science	
((AB=(alcohol* NEAR polic*) AND AB=(implement* OR enforc* OR monitor* OR compliance)) OR (AB=(alcohol* NEAR regulat*) AND AB=(implement* OR enforc* OR monitor* OR compliance)) OR (AB=(alcohol* NEAR strateg*) AND AB=(implement* OR enforc* OR monitor* OR compliance)) OR (AB=(alcohol* NEAR law*) AND AB=(implement* OR enforc* OR monitor* OR compliance)) OR (AB=(alcohol* NEAR legislation) AND AB=(implement* OR enforc* OR monitor* OR compliance))) NOT (AB=("non-alcohol*" OR “brief screening” OR screening OR “alcohol treatment”)) NOT SU= Zoology	2,656
AB=(taxes OR tax OR taxation OR pric* OR affordability OR "minimum pricing" OR "minimum unit pricing" OR "floor price") AND AB=(alcohol*) AND AB=(implement* OR enforc* OR monitor* OR compliance) NOT AB="non-alcohol*" NOT SU= Zoology	567
AB=(sale* OR “trading hour*” OR “intoxicat*” OR "on-premis*" OR "off-premis*" OR "minimum age" OR "minimum legal age" OR availabilit* OR retail* OR outlet* OR licen* OR "online deliver*" OR "online sale*")	2410

Search terms	Number of studies found
AND AB=(alcohol*) AND AB=(implement* OR enforc* OR monitor* OR compliance) NOT AB= “non-alcohol*” NOT SU= Zoology	
AB=(marketing OR promot* OR adverti* OR sponsor*) AND AB=(alcohol*) AND AB=(implement* OR enforc* OR monitor* OR compliance) NOT AB="non-alcohol*" NOT SU= Zoology	2,687
AB=(driv* OR “blood alcohol concentration”) AND AB=(alcohol*) AND AB=(implement* OR enforc* OR monitor* OR compliance) NOT AB= "non-alcohol*" NOT SU= Zoology	1889
Combined with all<br>#5 OR #4 OR #3 OR #2 OR #1<br>Refined by: PUBLICATION YEARS: (2021 OR 2005 OR 2020 OR 2004 OR 2019 OR 2003 OR 2018 OR 2002 OR 2017 OR 2001 OR 2016 OR 2000 OR 2015 OR 2014 OR 2013 OR 2012 OR 2011 OR 2010 OR 2009 OR 2008 OR 2007 OR 2006) AND DOCUMENT TYPES: (ARTICLE OR REPORT OR REVIEW OR THESIS DISSERTATION OR CASE REPORT OR CLINICAL TRIAL) AND LANGUAGES: (ENGLISH)	5,549
